# Symptom Clusters in Patients With Bone Metastases: A Sub-Analysis of Patients Reporting Exclusively Non-Zero BPI Scores

**DOI:** 10.4021/wjon394w

**Published:** 2012-02-19

**Authors:** Gemma Cramarossa, Janet Nguyen, Liying Zhang, Emily Chen, Luluel Khan, May Tsao, Cyril Danjoux, Elizabeth Barnes, Arjun Sahgal, Lori Holden, Flo Jon, Edward Chow

**Affiliations:** a Rapid Response Radiotherapy Program, Odette Cancer Centre, Sunnybrook Health Sciences Centre, University of Toronto, Canada

**Keywords:** Symptom cluster, Bone metastases, Brief pain inventory, Palliative radiotherapy

## Abstract

**Background:**

The use of different statistical methods and inclusion criteria when deriving symptom clusters in cancer patients are contributing factors in cluster inconsistencies across studies. Primary objective was to extract symptom clusters in a subgroup of patients reporting non-zero Brief Pain Inventory (BPI) scores at baseline, and to compare clusters with those identified in the total patient sample.

**Methods:**

Principal Component Analysis (PCA), Hierarchical Cluster Analysis (HCA) and Exploratory Factor Analysis (EFA) were performed on the non-zero subgroup and total patient sample to identify symptom clusters at baseline and 1, 2 and 3 months following radiotherapy.

**Results:**

At baseline, different symptom clusters were derived from the non-zero subgroup and the total patient population. Only PCA identified identical clusters. Over time, clusters extracted using the three statistical methods varied, with a few exceptions where the same clusters were extracted using two different methods at a specific time point. A complete consensus between all three methods was not noted at any time. The BPI, which is a short assessment tool, may lead to the extraction of oversimplified clusters. In addition, since this study analyzed results in the non-zero subgroup, clusters derived may be reflective of patients with poorer prognosis as these patients experienced all symptoms.

**Conclusion:**

Analyzing data compiled from all eligible consenting patients may not provide clinically relevant clustering among all symptoms in the assessment tool. The composition of symptom clusters varied with the inclusion of patients with zero symptom severity scores and with the statistical method employed.

## Introduction

Advanced cancer patients often develop bone metastases, with roughly 65 to 75% of prostate and breast cancer patients and 30 to 40% of lung cancer patients developing these lesions [1]. Substantial pain, hypercalcemia, spinal cord compression, and pathologic fractures are all complications that may result from bone metastases. These patients often experience multiple symptoms at one time that concurrently influence their quality of life in a multiplicative manner [2]. These symptom “clusters” consist of two or more interrelated symptoms that occur simultaneously [3, 4]. The discovery of symptom clustering has resulted in a shift from research in single symptoms to symptom clusters in order to comprehensively understand the symptom experience [5].

Symptom clusters can be identified through clinical means or through the use of several different statistical methods. Most symptom cluster studies include data from all eligible consenting patients, which may not provide an accurate depiction of clusters since some patients do not experience all symptoms included in the assessment (typically indicated by a score of zero). Since these patients are not representative of the entire range of symptoms available, symptom clusters derived may vary based on their exclusion. This becomes a significant issue when using brief assessment tools. The use of different statistical methods to derive symptom clusters is another concern, as it may largely contribute to inconsistencies in clusters across studies and makes comparisons difficult.

The primary objective of this study was to observe whether symptom cluster results differed in the subgroup of patients who reported only non-zero BPI (see abbreviations below) scores at baseline. The secondary objective was to determine whether symptom clusters varied when derived using three commonly utilized statistical methods.

## Patients and Methods

The dataset utilized in our previous study on symptom clusters, which was compiled from 348 bone metastases patients receiving radiation treatment, was also utilized in the current study in order to avoid inconsistencies due to varied assessment tools or sample population characteristics [6]. Ethics approval was obtained from Sunnybrook Health Sciences Centre and all patients provided informed consent. A trained research assistant administered questionnaires and collected all information in person at baseline and via telephone at follow ups. In addition, data on patient demographics, cancer history and analgesic consumption within the past 24 hours was recorded at the initial visit before radiation treatment was administered.

BPI questionnaires were administered during routine clinical assessment at baseline and 1, 2 and 3 months post radiation treatment. The BPI is the most frequently used multiple-item measure of pain in oncology [7]. It measures sensory and affective dimensions on an 11 point scale where 0 indicates no pain/does not interfere and 10 indicates worst imaginable pain/completely interferes for the pain scales and seven functional interference items respectively. The sensory dimension of pain intensity measures worst, average and current pain, however we only used worst pain in conjunction with the seven BPI functional interference items in order to search for symptom clusters, making up the eight item BPI. “Pain” was specific to the irradiated site for the purposes of this study. The affective dimension of functional interference measures: general activity, normal work, walking ability, mood, sleep, relations with other people and enjoyment of life.

### Statistical analysis

Symptom clusters were derived using Principal Component Analysis (PCA), Exploratory Factor Analysis (EFA) and Hierarchical Cluster Analysis (HCA).

PCA with varimax rotation was performed on the BPI items to examine any interrelationships between symptoms at each follow up time point. This analytical method groups variables (symptoms) together to form a “component” (cluster) by identifying which variables correlate with each other in a distinct pattern [8]. The highest loading factor score determines the assignment of symptoms to clusters. An eigenvalue greater than 1.0 and explaining almost 10% of the total variance was required to determine significant clusters. Cronbach’s alpha was calculated to determine the internal consistency and reliability of the clusters.

EFA is the most commonly used analytical method in oncology symptom cluster research. EFA differs from other analytical methods because it assumes symptoms in a cluster are correlated by latent factors, which bind the symptoms together [9]. Symptoms associated with one latent factor co-vary more closely with each other compared with symptoms associated with a different latent factor [10]. The factors (clusters) with eigenvalue greater than 1.0 were retained, meaning that approximately 10% of variance in the symptom is shared with the latent factor after controlling for the correlation between factors. The maximum likelihood method followed by the *varimax* orthogonal rotation method was then applied to approximately multivariate normal data to measure covariance between symptoms. Together these two methods identify and determine the items (symptoms) that belong to each cluster. The PROC FACTOR procedure in Statistical Analysis Software (SAS version 9.2) was conducted for this analysis. Cronbach’s alpha was calculated to measure the internal consistency of the clusters.

HCA extracted clusters using average linkage between groups. Similar entities were grouped together and each cluster was separated from other clusters [10]. HCA is also utilized to categorize groups of individuals with similar symptom profiles. Euclidian distances were used to measure the distance between symptom severities, which determined how clusters were formed. The PROC VARCLUS runs clusters on the basis of *centroid* components. The R^2^ values of each symptom with its own cluster and with its nearest cluster were calculated. The 1-R^2^ ratio is the ratio of one minus the value in the “Own Cluster” column to one minus the value in the “Next Closest” column. The lower the ratio, the more distinct the clusters are. HCA produces a dendrogram, or visual diagram, of clusters extracted. Symptoms that converge earlier on the dendrogram are more closely related than those that converge later.

### Subgroup analysis of patients with non-zero baseline BPI Scores

The “non-zero” subgroup of 138 patients included only patients who reported severity scores > 0 for all eight BPI items at baseline. Symptom clusters for this subgroup were identified using PCA, EFA and HCA and these clusters were subsequently compared with those identified in the total patient sample at all assessment time points. The temporal pattern of symptom clusters in the “non-zero” subgroup was also analyzed using the three statistical methods.

## Results

Symptom clusters in both patient samples were mainly observed when HCA was employed. The absence of clustering across time points in both samples was evident when the other two statistical methods were utilized. Symptom clusters derived using PCA, EFA and HCA at baseline in both patient samples were comparable. Depending on the analytical method employed, one to two clusters of two to seven symptoms each were extracted when looking at all assessment time points. Appendices display symptom clusters identified at each follow up for both patient groups.

### Baseline symptom clusters in the total patient sample versus non-zero subgroup

The symptom clusters extracted from the total patient sample and non-zero subgroup at baseline are summarized in Table 1.

**Table 1:** Baseline Symptom Clusters in the Total Patient Sample (T) and Non-Zero Subgroup (NZ)

Symptom	PCA		EFA		HCA	
	T (n = 348)	NZ (n = 138)	T (n = 348)	NZ (n = 138)	T (n = 348)	NZ (n = 138)
Worst Pain	Δ	Δ	Δ	Δ	Δ	—
General Activity	Δ	Δ	Δ	Δ	Δ	Δ
Walking Ability	Δ	Δ	Δ	Δ	Δ	Δ
Normal Work	Δ	Δ	Δ	Δ	Δ	Δ
Enjoyment of Life	Δ	Δ	Δ	Δ	Δ	Δ
Mood	Ο	Ο	---	Ο	Ο	Ο
Relations with others	Ο	Ο	---	Ο	Ο	Ο
Sleep	Ο	Ο	---	Ο	---	Ο

^a^PCA, Principal Component Analysis; EFA, Exploratory Factor Analysis; HCA, Hierarchical Cluster Analysis. ^b^Symptoms with corresponding symbols indicate they were in the same cluster.^c^Dash indicates the symptom was not present in any clusters.

PCA with varimax rotation was performed in our previous study on the BPI items in all 348 patients [6]. At baseline, two symptom clusters were extracted. Cluster 1 consisted of worst pain, general activity, walking ability, normal work and enjoyment of life. Cluster 2 included mood, relations with others and sleep. In the non-zero subgroup, PCA identified identical clusters that collectively account for 60.1% of the variance (Table 2). The Cronbach’s alpha for the non-zero subgroup are 0.80 and 0.74 for the first and second cluster, respectively.

**Table 2:** Factor Loadings and Final Communality From the Principal Component Analysis of BPI Symptoms in the Non-Zero Subgroup at Baseline (n = 138)

	Component 1	Component 2	Final communality
General activity	0.81	0.27	0.72
Normal work	0.81	0.25	0.72
Walking ability	0.72	0.07	0.53
Enjoyment of life	0.65	0.27	0.50
Worst Pain	0.57	0.18	0.36
Mood	0.23	0.82	0.72
Relations with others	0.30	0.79	0.72
Sleep	0.13	0.73	0.55
% of variance	46.3%	13.8%	
Cronbach’s alpha	0.80	0.74	
Eigenvalue	3.70	1.11	

^a^BPI, Brief Pain Inventory. ^b^Symptoms with bolded factor loadings were in the same cluster.

EFA identified a one cluster solution for the original total cancer patient sample which is identical to Cluster 1 derived using PCA (n = 348; Table 3). The Cronbach’s alpha was 0.88. In the non-zero subgroup, two clusters were derived (n = 129; Table 4). Cluster 1 was identical to the cluster identified in the total patient sample and using PCA for both sample populations at baseline. Cluster 2 was also identical to the cluster extracted using PCA at baseline in both patient samples. The Cronbach’s alpha for Clusters 1 and 2 in the non-zero subgroup were 0.80 and 0.74 respectively.

**Table 3:** Factor Loadings and Final Communality Determined Using Exploratory Factor Analysis in Total Patient Sample at Baseline (n = 348)

	Factor 1	Final communality
General activity	0.86	0.73
Normal work	0.77	0.59
Walking ability	0.67	0.45
Enjoyment of life	0.84	0.70
Worst Pain	0.64	0.41
Mood	0.65	0.42
Relations with others	0.54	0.30
Sleep	0.54	0.29
% of variance	100%	
Cronbach’s alpha	0.88	
Eigenvalue	9.58	

**Table 4:** Factor Loadings and Final Communality Determined Using Exploratory Factor Analysis in Non-Zero Subgroup at Baseline (n = 138)

	Factor 1	Factor 2	Final communality
General activity	0.80	0.26	0.71
Normal work	0.79	0.26	0.70
Walking ability	0.56	0.18	0.35
Enjoyment of life	0.62	0.31	0.37
Worst Pain	0.42	0.23	0.23
Mood	0.25	0.73	0.60
Relations with others	0.26	0.79	0.69
Sleep	0.23	0.46	0.27
% of variance	82.0%	17.9%	
Cronbach’s alpha	0.80	0.74	
Eigenvalue	8.49	1.86	

**Figure 1:**
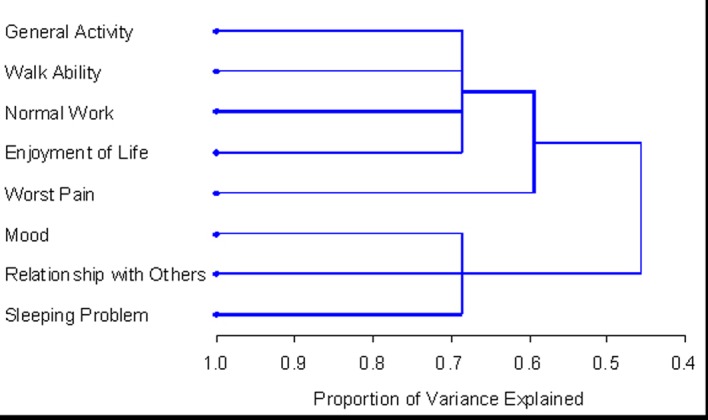
PROC TREE procedure generated dendrogram displaying three cluster solution and cluster hierarchy in 348 patients. One cluster (general activity, walk ability, normal work, and enjoyment) explained 53.9% of total variation; when adding worst pain, it explained up to 65.1% of total variation; when adding additional 3 items, the clusters explained up to 73.5% of total variation.

**Figure 2:**
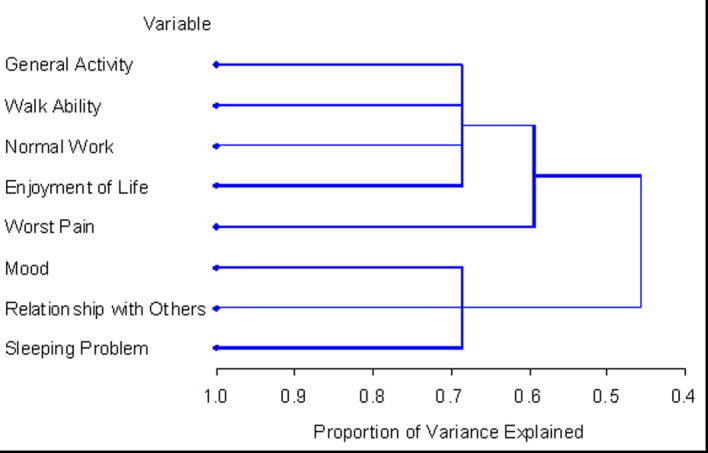
PROC TREE procedure generated dendrogram displaying two cluster solution and cluster hierarchy in 138 patients with non-zero record at baseline. One cluster (general activity, walk ability, normal work, and enjoyment) explained 45.6% of total variation; when adding worst pain, it explained up to 59.3% of total variation; when adding additional 3 items, the clusters explained up to 68.7% of total variation.

Using HCA, the number of symptom clusters identified from both patient groups was identical to that when using PCA; however the composition of these clusters varied slightly. Cluster 1 derived from HCA in the total patient sample was identical to the cluster derived using both EFA and HCA. Cluster 2 consisted of mood and relations with others. The final clusters solution in the total 348 patient sample explained 73.5% of the total variation. The dendrogram in Figure 1 is a visual representation of the clusters identified. The clusters extracted from the non-zero subgroup differed slightly. Cluster 1 included the same items with the exception of “pain” which was not present in any cluster. Cluster 2 was identical to the cluster derived using PCA in both patient samples and using EFA for the non-zero subgroup. The final clusters solution in the non-zero subgroup of 138 patients explained 68.7% of the total variation. Figure 2 displays the dendrogram which depicts the two symptom clusters identified.

### Temporal pattern of symptom clusters in the non-zero subgroup

Among the 138 eligible patients at baseline, there were 95 patients at week 4, 63 patients at week 8 and 53 patients at week 12. The temporal pattern of symptom clusters varied in the non-zero subgroup regardless of the statistical method employed, although the HCA method appeared to provide more stable clusters. Both PCA and EFA identified a general pattern of clustering, followed by no clustering at the next time point. HCA consistently identified two symptom clusters. Clusters derived using the three methods at each time point were never identical. The cluster findings over time are detailed in the Appendices.

While the temporal pattern of symptom clusters extracted using PCA and EFA were incongruent, the findings of both methods are in agreement regarding the instability of clusters over time. HCA derived one stable cluster across each time point.

### Consistency of symptoms cluster findings using PCA, EFA and HCA

There exist variations, albeit not very significant, in the quantity and composition of symptom clusters identified using the three statistical methods at each time point. Some exceptions include cluster findings using PCA which were identical to EFA at baseline in the non-zero subgroup, and at weeks 4 and 8 neither method identified any clusters in this subgroup. In addition, at 8 weeks PCA and EFA did not derive any clusters in the total patient sample. Although the entire cluster composition varied over time, some symptoms within clusters consistently occurred in conjunction. General activity, walking ability, normal work and enjoyment of life consistently occurred together despite the time point or statistical method employed. Worst pain frequently occurred with these symptoms as well. In addition, mood and relations with others often presented together regardless of the analytical method employed.

## Discussion

To our knowledge, this is the first study to derive symptom clusters using PCA, EFA and HCA over time in a subgroup of patients with bone metastases reporting exclusively non-zero severity scores for all BPI items. The clusters extracted were compared with clusters derived using the same three analytical methods in the total patient sample. As with many studies that determine symptom clusters, clusters were identified based on their severity ratings. A short series of steps were performed to empirically derive clusters. First, patients specified the presence and severity of each symptom in the assessment tool. Next, PCA, EFA and HCA were employed on the data collected from all patients and symptoms were clustered based on their severities at each specific time point.

Previous symptom cluster studies included all eligible consenting patients in the dataset. However, this inclusion criterion may distort the resulting symptom clusters. A specific symptom is absent in a patient if they indicate a severity score of zero, and the majority of the patient sample indicated a score of zero for at least one BPI item. The true definition of symptom clusters requires symptoms to co-occur, thus they must be present in order to be considered part of the same cluster. Thus, the inclusion of patients who did not experience all symptoms in the chosen assessment tool presents a concern because such data may not provide an accurate overview of the interrelationships between all eight BPI items.

In fact, the present study revealed incongruences between clusters identified in the total patient sample and the non-zero subgroup at baseline. Only the use of PCA identified identical clusters in both subgroups. Since disparities exist in cluster findings at baseline, it is reasonable to predict discrepancies over the course of treatment as well. Indeed, the only other time point at which identical clusters were extracted in both subgroups was at 4 weeks using HCA. This is indicative of the differences in symptom experiences between the two subgroups, thus the symptom experience of patients in the non-zero subgroup is not an accurate depiction of the experiences of the total population. These disparities convey the importance of screening patients based on their reported symptoms, especially when using brief assessment tools such as the BPI.

Varied cluster results when patient inclusion criteria are modified raise the concern of the necessity of a universal standard to determine when symptoms should be considered notable and included in cluster analysis. Clusters in the present study, as with many prior studies, were identified based on the interrelationships between symptoms as indicated by numeric severity scores [4]. The BPI was utilized in this study and allows patients to rate symptoms on a scale of 0 to 10. Symptoms may be further divided into mild, moderate and severe categories according to their numeric ratings. Previous studies identified a mild pain score as 1 to 4, moderate as 5 to 7 and severe as 8 to 10 [11]. It may also be proposed that mild symptoms, in addition to zero scores, should not be considered since symptoms only significantly affect a patient’s daily life as they become more severe. Symptom cluster disparities in patients reporting exclusively moderate or severe symptoms should be analyzed in future studies.

Employing several different statistical methods to identify symptom clusters is commonly debated as a source of inconsistency among studies. However, the literature indicates varying study results about this concern. There are a few previous studies that have noted similar, albeit not identical, symptom cluster results when different analytical methods are utilized [12-14]. Our present study reveals somewhat similar, however very rarely identical cluster findings. Thus, the disparities demonstrate that the statistical method directly influences the resulting clusters, which limits clinicians’ ability to apply findings to universally improve treatment and symptom management. An optimal statistical method must be agreed upon by clinicians and statisticians in order for future symptom cluster studies to be objectively compared.

Limitations of the present study include the use of the BPI, which is a short assessment tool, thus potentially leading to the extraction of oversimplified clusters. Cancer patients experience a broad range of symptoms and functional interferences, and the eight item BPI may not comprehensively assess the entire range of a patient’s functional status. Additional clusters may be identified upon the addition of more symptoms. Also, since the primary cancer of each patient varies, disparities in symptom experience may occur since different cancer populations may experience slightly different symptoms. In addition, this study analyzed results in the non-zero subgroup, which may be reflective of patients with poorer prognosis as these patients likely experienced more symptoms than the general bone metastases population. The findings of our study should be interpreted with these considerations in mind.

Symptom cluster research is complex and ongoing in oncology. Our present study identified two concerns hindering the identification of the most clinically relevant clusters. Inclusion criteria based on symptom severity requires further analysis and clarification. Further studies comparing statistical methods are warranted to determine a universal statistical method to employ in order to eliminate inconsistency. Accurate symptom cluster studies are promising for developing innovative cancer treatment strategies. Previous studies reporting the prognostic effect of multiple concurrent symptoms on functional status and quality of life support this notion [5, 15, 16].

## Abbreviations

BPI: Brief Pain Inventory
PCA: Principal Component Analysis
HCA: Hierarchical Cluster Analysis
EFA: Exploratory Factor Analysis
